# Diminished or inversed dose-rate effect on clonogenic ability in Ku-deficient rodent cells

**DOI:** 10.1093/jrr/rraa128

**Published:** 2020-12-29

**Authors:** Hisayo Tsuchiya, Mikio Shimada, Kaima Tsukada, Qingmei Meng, Junya Kobayashi, Yoshihisa Matsumoto

**Affiliations:** Laboratory for Advanced Nuclear Energy, Institute of Innovative Research, Tokyo Institute of Technology, 2-12-1 Ookayama, Meguro-ku, Tokyo 152-8550 Japan; Laboratory for Advanced Nuclear Energy, Institute of Innovative Research, Tokyo Institute of Technology, 2-12-1 Ookayama, Meguro-ku, Tokyo 152-8550 Japan; Laboratory for Advanced Nuclear Energy, Institute of Innovative Research, Tokyo Institute of Technology, 2-12-1 Ookayama, Meguro-ku, Tokyo 152-8550 Japan; Department of Interdisciplinary Environment, Graduate School of Human and Environmental Sciences, Kyoto University, Yoshidanihonmatsucho, Sakyo-ku, Kyoto 606-8501 Japan; Radiation Biology Center, Graduate School of Biostudies, Kyoto University, Yoshida-Konoecho, Sakyo-ku, Kyoto 606-8501 Japan; Laboratory for Advanced Nuclear Energy, Institute of Innovative Research, Tokyo Institute of Technology, 2-12-1 Ookayama, Meguro-ku, Tokyo 152-8550 Japan

**Keywords:** dose-rate effect, DNA double-strand break repair, non-homologous end joining, Ku, DNA-PKcs

## Abstract

The biological effects of ionizing radiation, especially those of sparsely ionizing radiations like X-ray and γ-ray, are generally reduced as the dose rate is reduced. This phenomenon is known as ‘the dose-rate effect’. The dose-rate effect is considered to be due to the repair of DNA damage during irradiation but the precise mechanisms for the dose-rate effect remain to be clarified. Ku70, Ku86 and DNA-dependent protein kinase catalytic subunit (DNA-PKcs) are thought to comprise the sensor for DNA double-strand break (DSB) repair through non-homologous end joining (NHEJ). In this study, we measured the clonogenic ability of Ku70-, Ku86- or DNA-PKcs-deficient rodent cells, in parallel with respective control cells, in response to high dose-rate (HDR) and low dose-rate (LDR) γ-ray radiation (~0.9 and ~1 mGy/min, respectively). Control cells and murine embryonic fibroblasts (MEF) from a severe combined immunodeficiency (scid) mouse, which is DNA-PKcs-deficient, showed higher cell survival after LDR irradiation than after HDR irradiation at the same dose. On the other hand, MEF from Ku70^−/−^ mice exhibited lower clonogenic cell survival after LDR irradiation than after HDR irradiation. XR-V15B and *xrs*-5 cells, which are Ku86-deficient, exhibited mostly identical clonogenic cell survival after LDR and HDR irradiation. Thus, the dose-rate effect in terms of clonogenic cell survival is diminished or even inversed in Ku-deficient rodent cells. These observations indicate the involvement of Ku in the dose-rate effect.

## INTRODUCTION

For sparsely ionizing radiations, such as X-rays and γ-rays, the biological effect of a given dose is generally reduced as the dose rate is reduced. This phenomenon, which is termed ‘the dose-rate effect’, is observed in a wide range of biological systems, including human epidemiology, animal models and cultured cells, and is emphasized greatly in cancer radiotherapy and radiological protection [1, 2]. It is thought that the dose-rate effect is due to the repair of DNA damage during irradiation but the precise mechanism of the dose-rate effect remains to be clarified.

Among various types of DNA damage, DNA double-strand breaks (DSB) are considered the most serious type. In eukaryotes, including humans, DSBs are repaired mainly through non-homologous end joining (NHEJ) and homologous recombination (HR) [3]. NHEJ operates throughout the cell cycle, while HR is limited to late S and G2 phases in higher eukaryotes, including humans, as sister chromatid is required as the template for HR [3]. Mammalian cells, including human cells, rely on NHEJ to repair DSBs in G1, where they spend most of the time in the cell cycle. Even in late S and G2 phases, the majority, i.e. 70–80%, of DSBs are repaired through NHEJ [3]. In NHEJ, a ternary complex of Ku70, Ku86 and DNA-dependent protein kinase catalytic subunit (DNA-PKcs) is thought to act as the molecular sensor for DSBs [3]. Cells or animals lacking any of these three molecules exhibit elevated sensitivity to ionizing radiation and defective V(D)J recombination, leading to immunodeficiency [3]. This study aimed to examine possible involvement of Ku70, Ku86 and DNA-PKcs in the dose-rate effect in terms of clonogenic cell survival.

## MATERIALS AND METHODS

### Cells

In this study, four NHEJ-deficient rodent cell lines, i.e. Ku70^−/−^, SCID, XR-V15B and *xrs*-5 cells, and their respective control cell lines were used.

SCID and CB17 are murine embryonic fibroblasts (MEF) cell lines established from severe combined immunodeficiency (scid) mice, harboring mutation in the gene encoding DNA-PKcs [4–9], and the parental CB17 mice, respectively [10], and were obtained from Dr Kenshi Komatsu at Kyoto University.

Ku70^−/−^ is an MEF cell line established from Ku70 knockout mice [11, 12] and was obtained from Dr Frederick W. Alt at Harvard University, USA. As the control for Ku70^−/−^, murine Ku70 cDNA was introduced into Ku70^−/−^ and the stable transformant, hereafter denoted Ku70^−/−^+Ku70, was obtained [13].

XR-V15B and *xrs*-5 are Ku86-deficient mutants derived from Chinese hamster lung fibroblast cell line V79B, which is a subline of V79, and Chinese hamster ovary cell line CHO-K1, respectively [14–17]. XR-V15B, V79, *xrs*-5 and CHO-K1 were obtained from American Type Culture Collections (ATCC).

These cells were cultured in Dulbecco’s Modified Eagle Medium (DMEM) containing 4.5 g/L glucose supplemented with 10% v/v fetal bovine serum, 100 μg/ml streptomycin and 100 units/ml penicillin and 1% v/v non-essential amino acid cocktail, at 37°C in a humidified atmosphere containing 5% CO_2_. For Chinese hamster cells, DMEM/Ham’s F12 (1:1) medium supplemented with 10% v/v new born calf serum, 100 μg/ml streptomycin and 100 units/ml penicillin was also used. Fetal bovine serum and new born calf serum were purchased from HyClone (Logan, UT, USA) and other reagents were purchased from Nacalai Tesque (Kyoto, Japan). Cells were prepared for experiments so that they were in the logarithmic phase at the start of irradiation.

### Irradiation

For high dose-rate (HDR) irradiation, cells were irradiated with γ-ray from a ^60^Co source (222 TBq as of February 2010) in Tokyo Institute of Technology or with that from a ^137^Cs source (133.2 TBq as of December 1998) in Kyoto University at ambient temperature. The dose rates were 0.6–6 Gy/min, depending on the desired dose, for the former and 0.855–0.911 Gy/min for the latter. For low dose-rate (LDR) irradiation, cells were kept in a CO_2_ incubator, which was set at ~1 m distance from the ^137^Cs source (1.85 TBq as of November 1995) until the desired dose was achieved (1 or 2 days for 1.5 and 3 Gy, respectively). The dose rate was 1.0–1.1 mGy/min.

Note that the United Nations Scientific Committee on the Effects of Atomic Radiation (UNSCEAR) in its 2010 report defined LDR as 0.1 mGy per min, averaged over 1 h, or less [18]. It says, nevertheless, that the objective is to provide evidence-based estimates of the risks to human health from exposure to low doses and low dose-rates of radiation and also that different values are used to define low dose and low dose-rate for other purposes. The dose rate in this study is 10–11 times higher than the UNSCEAR definition, but is three orders of magnitude lower than generally used dose rate in radiation biology and within the range of low dose-rate in radiotherapy, including brachytherapy. Therefore, we refer to this condition as LDR in this study.

### Cell survival assay

Before irradiation, cells were counted, diluted and plated onto 10 cm tissue culture dishes in triplicate. After 8–15 days, colonies were stained with crystal violet and those consisting of >50 cells were counted. Plating efficiency was calculated as the number of colonies divided by the number of plated cells. Surviving fraction was calculated as the plating efficiency of irradiated cells divided by that of unirradiated cells. To obtain the D_10_, the dose to give a surviving fraction of 0.1, for HDR ^60^Co irradiation, the survival curves were fitted to an linear-quadratic (LQ) model equation, *i.e.*  *S* = exp(−α*D* − β*D*^2^), where *S* and *D* are surviving fraction and radiation dose, respectively, and α and β are coefficients. Statistical significance was tested by one-sided t-test, pairing the results from the same experiment.

### Cell cycle analysis

To analyze the cell cycle, cells were labeled with 5-ethynyl-2′-deoxyuridine (EdU) and Alexa Fluor 488 azide through Click reaction using a Click-iT EdU Imaging kit (C10337, Life Technologies, Eugene, OR, USA) and then stained with propidium iodide using a Cell Cycle Phase Determination Kit (10009349, Cayman Chemicals, Ann Arbor, MI, USA) according to the manufacturer’s protocol. Briefly, cells were cultured in the presence of 5 μM EdU for 30 min at 37°C. Cells were detached from the culture dish by treatment with 0.5 g/L trypsin and 0.2 mM EDTA and harvested using the culture medium. All the following processes were carried out on ice or at 4°C. The cells were then precipitated by centrifugation at 300 g for 4 min and resuspended in Ca^2+^, Mg^2+^-free Dulbecco's phosphatebuffered saline (D-PBS(−)) containing 0.1% w/v bovine serum albumin (BSA/PBS). After centrifugation at 300 g for 4 min, the cells were resuspended in 0.5 mL of methanol and kept at −20°C for >2 h for fixation. The cells were then centrifuged at 300 g for 4 min and resuspended in 0.85 mL of BSA/PBS. After centrifugation at 300 g for 4 min, the cells were resuspended in 50 μL of Click-it reaction cocktail containing Alexa Flour 488 azide and kept at ambient temperature for 1 h. Hereafter cells were protected from light by aluminum foil. After addition 150 μL of BSA/PBS, the cells were centrifuged at 300 g for 4 min and resuspended in 50 μL of D-PBS(−) containing 0.02% w/v sodium azide (Nacalai Tesque), 0.02% v/v RNase A solution and 0.01% v/v propidium iodide reagent (the components of Cell Cycle Phase Determination Kit from Cayman Chemicals). After keeping at ambient temperature for 1 h, the cell suspension was supplemented with 250–400 μL of BSA/PBS for adjustment of the volume and was subjected to flowcytometric analysis using Cell Lab Quanta SC (Beckman Coulter).

## RESULTS

**Fig. 1.**
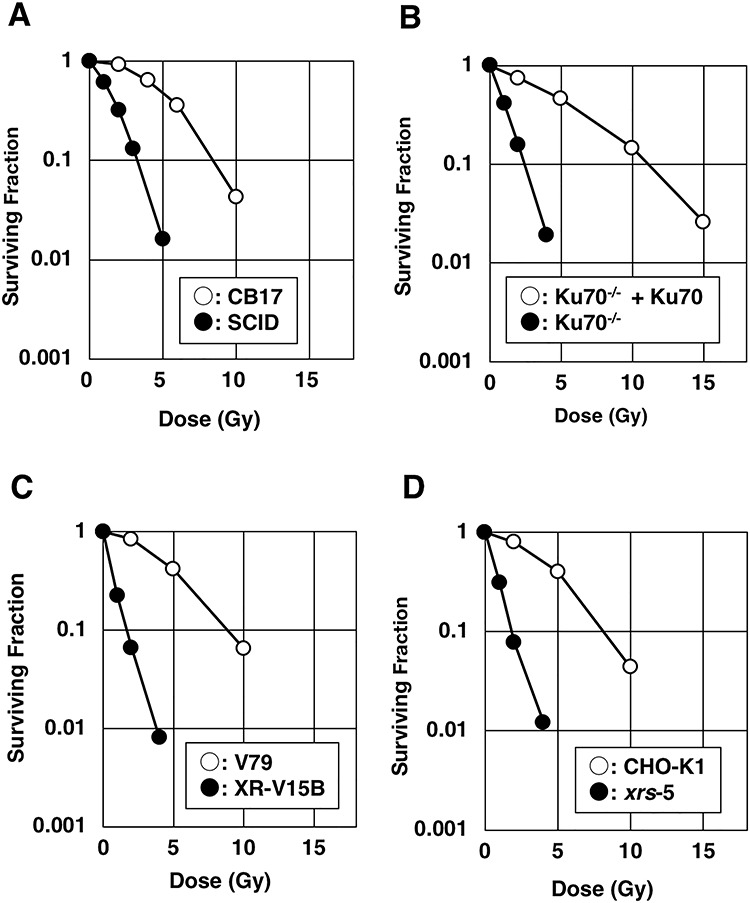
Clonogenic cell survival of NHEJ-deficient rodent cells and respective control cells for HDR γ-ray from a ^60^Co source. (**A**) SCID (DNA-PKcs-deficient) and CB17. (**B**) Ku70^−/−^ and Ku70^−/−^+Ku70. (**C**) XR-V15B (Ku86-deficient) and V79. (**D**) *xrs*-5 (Ku86-defiient) and CHO-K1.

Figure 1 shows the clonogenic cell survival of the eight cell lines after HDR ^60^Co γ-ray irradiation. Ku70-, Ku86- or DNA-PKcs-deficient cells showed higher radiosensitivity than respective control cells. D_10_ is shown in Table 1.

**Table 1:** D_10_ for HDR γ-rays from a ^60^Co source

Cell	D_10_ (Gy)
SCID	3.3
CB17	8.6
Ku70^−/−^	2.4
Ku70^−/−^ + Ku70	11.1
V79	9.0
XR-V15B	1.6
CHO-K1	8.4
*xrs*-5	1.9

**Fig. 2.**
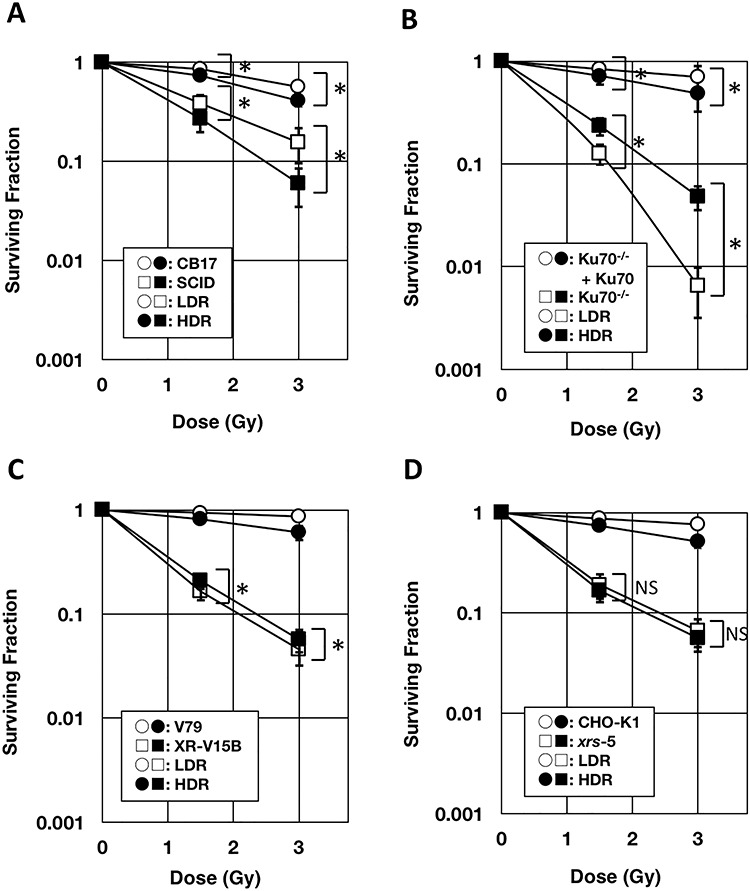

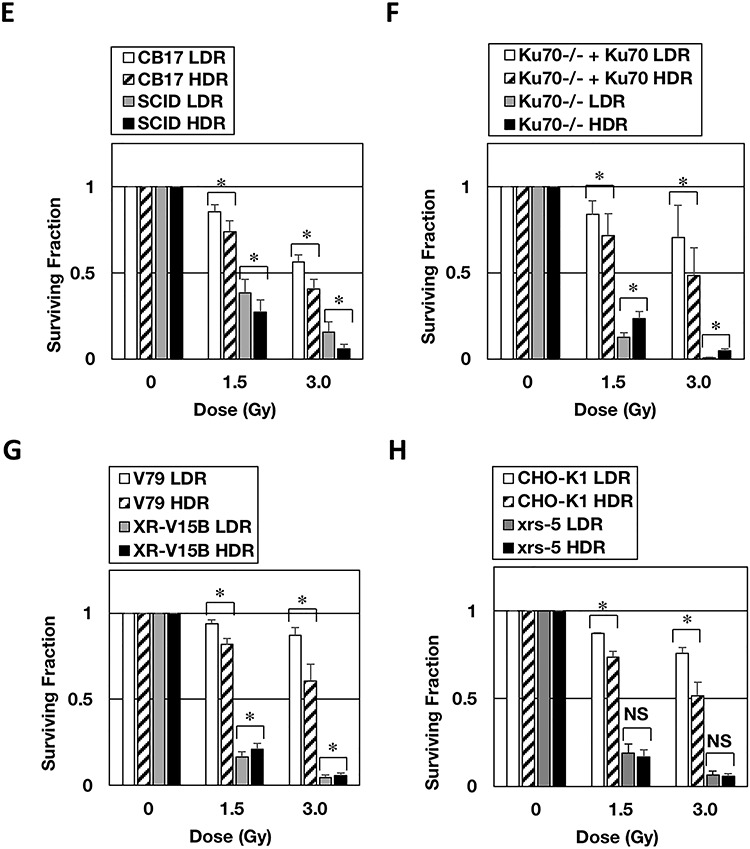
Clonogenic cell survival of NHEJ-deficient rodent cells and respective control cells for HDR and LDR γ-rays from a ^137^Cs source. (**A**, **E)** SCID (DNA-PKcs-deficient) and CB17. (**B**, **F**) Ku70^−/−^ and Ku70^−/−^+Ku70. (**C**, **G**) XR-V15B (Ku86-deficient) and V79. (**D**, **H**) *xrs*-5 (Ku86-deficient) and CHO-K1. The surviving fraction is displayed in logarithmic scale (A–D) and linear scale (E–H). Each symbol or column represents the average of 3–5 repeated experiments. Error bars represent the standard errors of the mean. ^*^P<0.05; NS, not statistically significant.

Figure 2 shows the clonogenic cell survival after ^137^Cs γ-ray irradiation at HDR (0.855–0.911 Gy/min) or LDR (1.0–1.1 mGy/min). D_10_ for HDR and LDR ^137^Cs γ-ray irradiation is shown in Table 2. We continued LDR irradiation of CB17, Ku70^−/−^+Ku70 and V79 cells until the endpoint, *i.e.* the time of fixation of the cells for colony counting, but the surviving fraction remained >0.5. Therefore, the dose delivered in the meantime is shown as the lower limit of D_10_. Control cells and DNA-PKcs-deficient SCID showed higher clonogenic cell survival after LDR irradiation than after HDR irradiation (Figs 2A and E). In contrast, Ku70^−/−^ showed significantly lower clonogenic cell survival after LDR irradiation than after HDR irradiation (Figs 2B and F). Ku86-deficient cells, XR-V15B and *xrs*-5, exhibited mostly identical clonogenic cell survival after LDR and HDR irradiation, although XR-V15B showed slightly lower clonogenic cell survival after LDR than after HDR irradiation (Figs 2C and G). Thus, the dose-rate effect was diminished or even inversed in Ku-deficient rodent cells. These observations indicate the involvement of Ku in the dose-rate effect.

**Table 2:** D_10_ for HDR and LDR γ-rays from a ^137^Cs source

Cell	HDR D_10_ (Gy)	LDR D_10_ (Gy)
SCID	2.5	3.2^a^
CB17	ND^b^	>19.0^c^
Ku70^−/−^	2.3	1.6
Ku70^−/−^ + Ku70	ND^b^	>19.0^c^
V79	ND^b^	>12.5^c^
XR-V15B	2.3	2.0
CHO-K1	ND^b^	ND^b^
*xrs*-5	2.1	2.3

^a^Obtained by extrapolation of the survival curve shown in Fig. 2A.

^b^Not determined.

^c^Lower limit is shown because the surviving fraction (S.F.) was >0.1 when LDR-irradiation was continued until the endpoint, i.e. the fixation of the cells for colony counting.

We analyzed the cell cycle distribution of Ku70^−/−^, XR-V15B, SCID and their respective control cells after LDR irradiation for 1.5 and 3.0 Gy (23 and 46 h at a dose rate of 1.1 mGy/min) by flowcytometry, staining DNA with propidium iodide and labeling S phase cells with EdU (Figs S1 and S2, see online supplementary material). Ku70^−/−^ showed a marked increase in G2/M phase cells and a decrease in S phase cells after LDR irradiation, indicating that the G2/M checkpoint was strongly activated. XR-V15B showed an increase in G2/M phase cells, albeit to a lesser extent than Ku70^−/−^. A modest increase in G2/M phase cells was observed in Ku70^−/−^+Ku70 and SCID. V79 and CB17 did not show an obvious increase in G2/M phase cells.

## DISCUSSION

The current study sought to evaluate the roles of Ku70, Ku86 and DNA-PKcs in the dose-rate effect in terms of clonogenic ability. Tomita *et al*. evaluated the roles of Ku70, DNA-PKcs, Rad51B and Rad54, in cell proliferation under LDR culture conditions (1 mGy/h, which is ~60 times lower than the LDR conditions here) [19]. Ku70- or DNA-PKcs-deficient chicken DT40 cells as well as DNA-PKcs-deficient human glioma M059J cells exhibited a greater extent of growth delay under LDR culture conditions than respective normal control cells or HR-deficient cells, indicating the importance of NHEJ in cell proliferation under LDR irradiation. Kreder *et al*. measured the effects of pulsed LDR irradiation (p-LDR, 0.1 Gy/pulse at 9.16 cGy/min with an interval of 4 min 52 s between pulses, giving 1 Gy/h on average, which is ~15 times higher than the LDR condition here) by colony formation assay [20]. Mutant cells defective in NHEJ or HR did not show substantial difference in cell survival after p-LDR and HDR irradiation, indicating the importance of NHEJ and HR in cell survival under LDR irradiation. The dose rate in this study is different from dose rates in these studies by more than one order of magnitude. The dose-rate effect is thought to result from (i) the repair of sublethal damage, (ii) redistribution in the cell cycle, and (iii) cell proliferation. While repair and proliferation will increase cell survival, redistribution in the cell cycle will reduce it. In general, NHEJ is thought to contribute to ~ 80% of DSB repair in G1 phase and ~70% in G2 phase and to repair DSBs with a half-life of 1–2 h [3], which is shorter than the time for exposure in all of the above three conditions. The involvement of Ku in sublethal damage repair was demonstrated by split-dose experiments, in which the interval between two doses is 2–4 h [21]. In the study by Kreder *et al*., the time required for irradiation would be shorter than the doubling time [20]. Therefore, the influence of redistribution and proliferation might be more significant in the conditions of the current study and the study by Tomita *et al*. than in the study by Kreder *et al*. In the condition of the study by Tomita *et al*., the total dose obtained in 8–15 days, i.e. the endpoint of colony formation assay in this study, is 192–360 mGy, where the primary term of the LQ equation is dominant. In this dose range, the dose rate effect is expected to be smaller than in higher doses such as 1.5 and 3.0 Gy. The colony formation assay is influenced by cell proliferation when the time for irradiation is long compared to the length of cell cycle. The ability of the initially existing cell to form a colony, which is measured in the colony formation assay, is overestimated due to the increased number of target cells to be inactivated. To compensate for this, it is necessary to take both cell division and cell death into consideration. We are now doing another study to simulate computationally cell proliferation under LDR irradiation.

In the current study, although both Ku and DNA-PKcs are involved in DSB repair through NHEJ, the impacts of their defects on the dose-rate effect were considerably different. While the dose-rate effect was diminished or even inversed in Ku-deficient cells, the normal dose-rate effect was observed in DNA-PKcs-deficient SCID cells. DNA-PKcs mutation in *scid* mice (c.T12,138A, p.Y4,046X) results in the loss of only 83 amino acids at the carboxy terminus-retaining kinase catalytic domain [7–9]. Nevertheless, this mutation results in greatly reduced, barely detectable, expression of DNA-PKcs [4, 7]. Moreover, the phenotype of mice with targeted disruption of DNA-PKcs was indistinguishable from that of *scid* mice [22, 23]. Considering this, the observed dose-rate effect in SCID is not likely due to residual function of DNA-PKcs but rather to Ku-dependent NHEJ in the absence of DNA-PKcs. Whereas Ku-deficient cells are completely devoid of signal joint formation and coding joint formation in V(D)J recombination [14, 16], DNA-PKcs-deficient cells are at least partially competent for signal joint formation [4–6, 22, 23].

There was also some difference in the dose-rate effect among Ku-deficient cells. While the dose-rate effect was diminished in two Ku86-deficient Chinese hamster cells, it was inversed in murine Ku70-deficient cell. Ku70 and Ku86 become highly unstable and mostly undetectable in the absence of each other [15, 17]. Ku70^−/−^ mice and Ku86^−/−^ mice show similar phenotype but still show differences. Ku86^−/−^ mice show more severe immunological defects than Ku70^−/−^ mice [12, 24–26]. Additionally, Ku70 is shown to bind to Bax to antagonize apoptosis [27]. However, the difference in the dose-rate effect cannot be simply attributed to such functional differences between Ku70 and Ku86. Residual functionality of Ku might be different among the Ku-deficient cell lines used here. Ku70^−/−^ knockout cells were generated through removal of exons 4 and 5 and the potential transcript would encode a truncated protein consisting of only 64 out of 608 amino acids [11, 12]. XR-V15B was shown to express Ku86 mRNA with a deletion of 138 nt, resulting in the in-frame deletion of 46 amino acids from codons 372–417 of 732 amino acids [15]. Another Ku86 allele of XR-V15B might have been silenced, as a natural revertant of XR-V15B was shown to express wild-type Ku86 mRNA [15]. *xrs*-5 showed greatly reduced, barely detectable, expression of Ku86 mRNA, although the mutation has not been found [17]. Finally, difference in the dose-rate effect could be due to differences in cellular characteristics. It is especially of note that p53 is reported to be non-functional in V79 and CHO-K1 cells [28, 29], possibly abrogating cell cycle checkpoints and/or apoptosis. The p53 status of Ku70^−/−^, Ku70^−/−^+Ku70, SCID and CB17 was established from mice with wild-type p53, but p53 function might have been abrogated during the establishment of the cell line by SV40 transfection. Mitchell *et al*. reported that human cervical carcinoma HeLa cells showed decreased cell survival with decreasing the dose rate from 1.54 to 0.37 Gy/h [30]. It was considered due to the G2/M checkpoint, which would arrest the cells in the most radiosensitive phase in the cell cycle: LDR irradiation resulted in longer stay and larger dose received at the G2/M boundary. In this regard, Ku70^−/−^ showed more marked accumulation in G2/M phases after LDR irradiation than XR-V15B in this study. Thus, the sensitivity of Ku70^−/−^ for LDR irradiation might have been augmented by redistribution, accumulating in G2/M phases.

In conclusion, we showed that the dose-rate effect in terms of clonogenic ability is diminished or even inversed in Ku-deficient cells. This observation indicated the involvement of Ku in the dose-rate effect and might also suggest a possible strategy for potentiating cancer treatment using LDR, *e.g.* brachytherapy, by targeting Ku.

## Supplementary Material

FigureS1_rraa128

FigureS2_2_r2_rraa128
