# Supplementary figures and images for: Diminished or inversed dose-rate effect on clonogenic ability in Ku-deficient rodent cells
Source: J Radiat Res. 2020 Dec 29;62(2):198–205. doi: 10.1093/jrr/rraa128 (PMC7948855; doi:10.1093/jrr/rraa128)

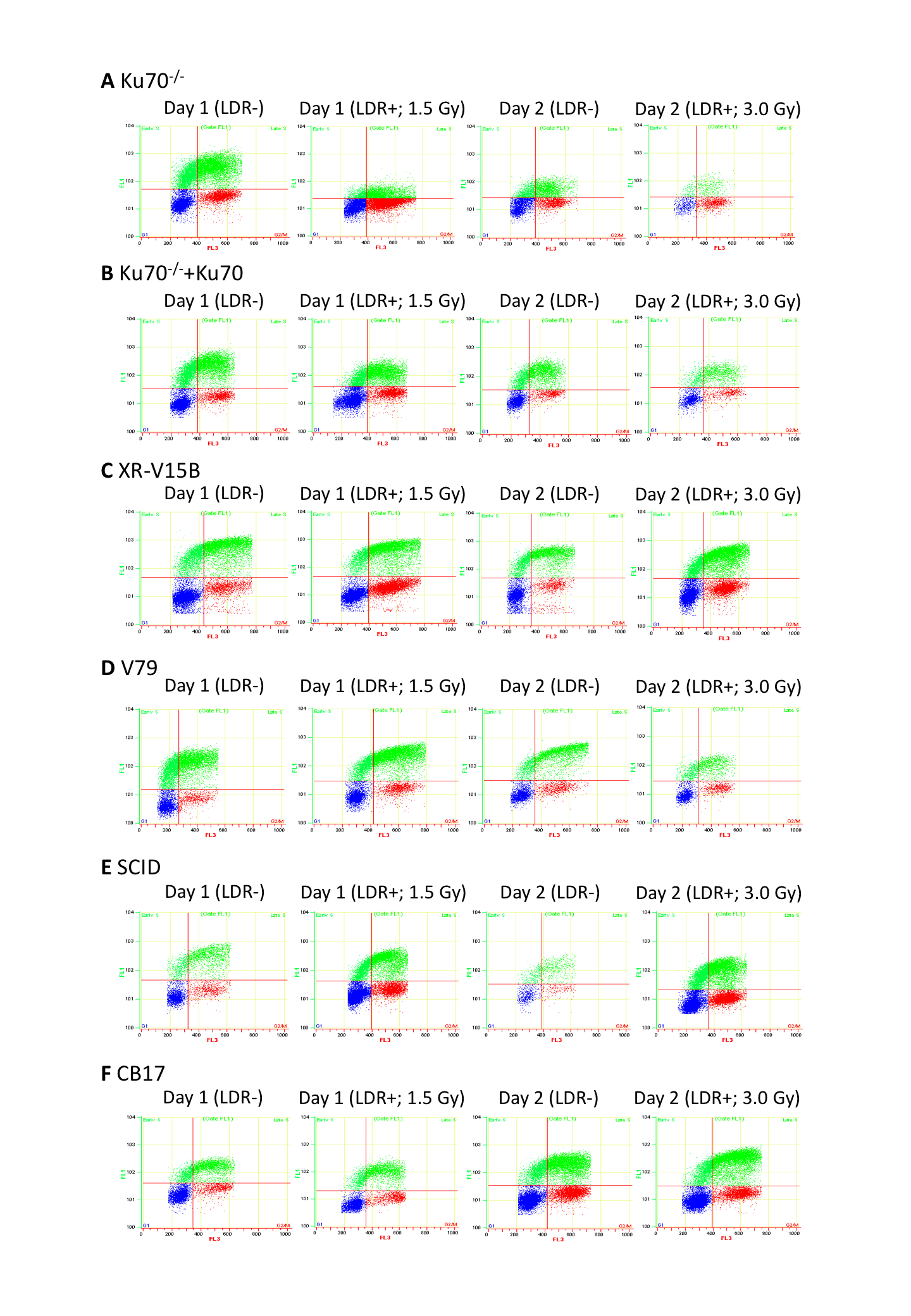

Supplement: FigureS1_rraa128 [file figures1_rraa128.png]

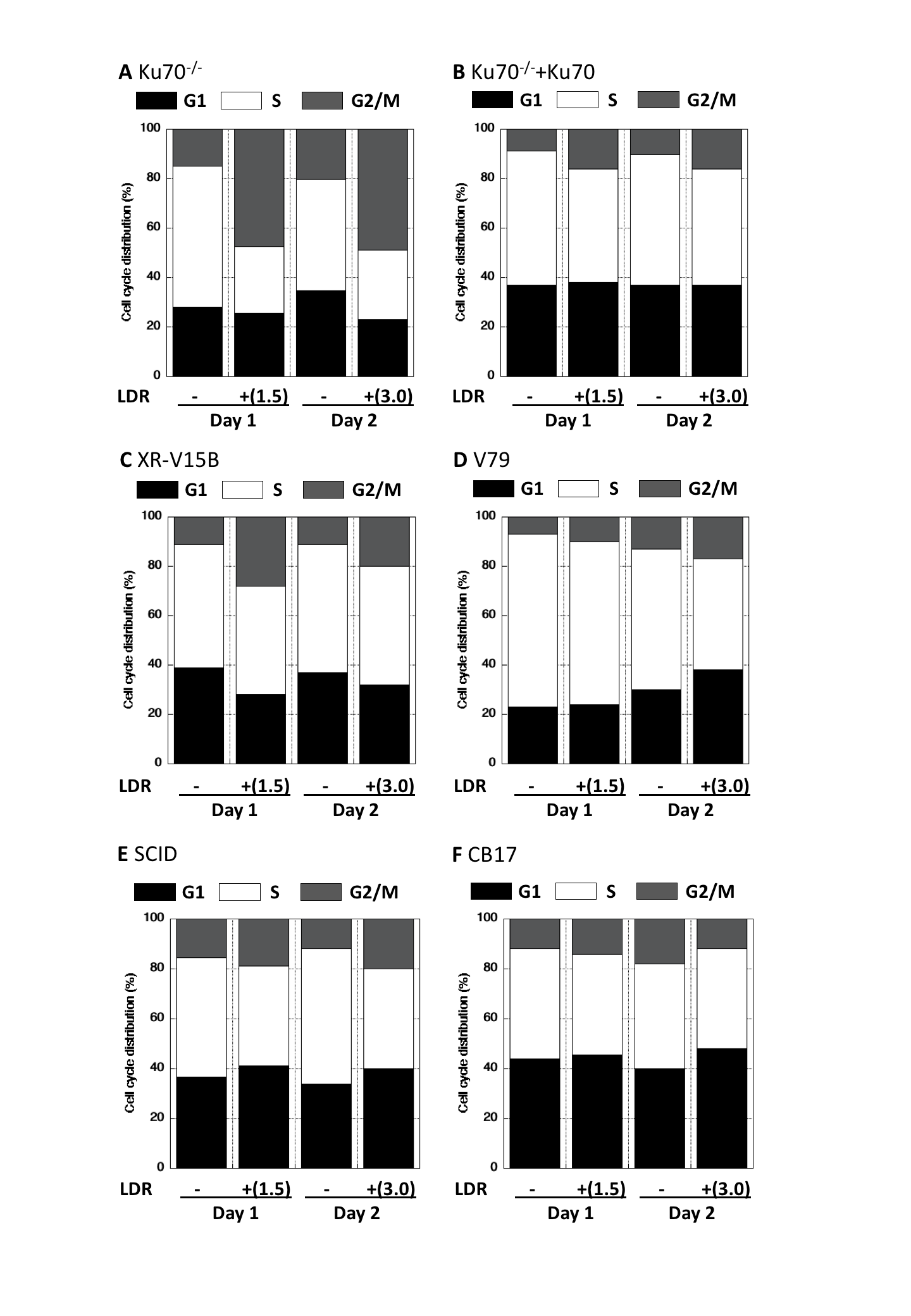

Supplement: FigureS2_2_r2_rraa128 [file figures2_2_r2_rraa128.png]
